# Comment on ‘The Life‐Course Changes in Muscle Mass Using Dual‐Energy X‐Ray Absorptiometry: The China BCL Study and the US NHANES Study’ by Wang Et Al.

**DOI:** 10.1002/jcsm.13591

**Published:** 2024-09-30

**Authors:** Qing Lan, Long Guo, Zhifan Xiong

**Affiliations:** ^1^ Department of Gastroenterology, Liyuan Hospital Tongji Medical College of Huazhong University of Science and Technology Wuhan China; ^2^ Department of Urology The Second Affiliated Hospital of Nanchang University Nanchang China

Dear Editor,

We read with great interest the recently published article by Wang et al. [1] in your esteemed journal, titled ‘The life‐course changes in muscle mass using dual‐energy X‐ray absorptiometry: The China BCL study and the US NHANES study’. The study [1] is commendable for its large sample size, comprehensive age range and inclusion of multiple ethnic groups, providing valuable insights into the muscle mass development trajectory across the life course in the Chinese population and its comparison with US populations. The study's [1] primary strength lies in its use of dual‐energy X‐ray absorptiometry (DXA) to accurately quantify muscle mass, and the application of the generalized additive model for location scale and shape (GAMLSS) to generate age‐ and sex‐specific percentile curves. These methodologies offer a robust framework for understanding muscle mass trajectories at different life stages, which is crucial for the early diagnosis and intervention of sarcopenia.

However, we believe that considering additional factors could further enhance the interpretation of these findings and provide valuable direction for future research.

First, the data collected span a decade (2013 to 2023) [1], with a large and diverse sample. However, the potential impact of temporal effects, such as improvements in healthcare and socio‐economic conditions, may introduce heterogeneity into the results. We suggest stratifying the data by time periods to identify and understand trends over different years, which could clarify the influence of temporal effects and help explain any time‐related variations. Although the study covers nine cities in China [1], the sample may not fully represent the entire Chinese population, particularly in rural areas and other unrepresented cities. Expanding the geographic coverage in future studies could provide a more comprehensive reflection of the diverse regions and populations across China, thereby enhancing the generalizability of the findings.

Second, while the study employs rigorous inclusion and exclusion criteria, which is commendable, certain potential factors such as dietary habits [2], physical activity levels [3], psychological factors [4] and socio‐economic status [5] have not been fully explored. For instance, participants' mental health could affect muscle mass through behavioural and physiological mechanisms like appetite changes and hormonal fluctuations. Socio‐economic status, including income levels, education and occupation, might also indirectly influence muscle mass by affecting participants' diet, exercise and overall health status. Future research could consider excluding individuals with extreme dietary habits, extreme physical activity levels and abnormal psychological factors or include these potential factors as covariates in stratified analyses. A deeper exploration of these factors would contribute to a better understanding of the observed differences and provide stronger support for the reliability of the research findings.

Sarcopenia is a critical indicator of poor health, and as social workers, we play a key role in its prevention and management, helping individuals and communities prioritize muscle health to improve overall quality of life [6]. Through both online and offline campaigns, we can educate the public about sarcopenia, including its symptoms, risk factors and preventive measures. For those whose quality of life or mobility is impaired due to sarcopenia, providing psychological and emotional support is essential in helping them cope with the emotional challenges posed by the disease and maintaining a positive mindset. Collaborating with healthcare professionals to screen and assess high‐risk groups for sarcopenia, and offering personalized intervention recommendations, is crucial. Assisting clients in accessing healthcare resources, dietitians and physical therapists ensures they receive comprehensive support and treatment. Therefore, it is imperative that governments, stakeholders, civil society, healthcare providers and individuals work together to create a collaborative mechanism to promote the prevention and management of sarcopenia. Such multistakeholder cooperation can integrate resources, share knowledge and provide holistic support, ensuring that everyone receives the necessary help and services to effectively address this public health challenge.

In conclusion, Wang et al.'s study makes a significant contribution to our understanding of muscle mass development across the life course in different populations. We look forward to future research that further explores and addresses these limitations, deepening our understanding of the complex factors influencing muscle mass and revealing their potential impact on the health of diverse populations.

Yours sincerely,

Qing Lan

## Conflicts of Interest

The authors declare no conflicts of interest.

## References

[1] X. Wang , L. Gao , J. Xiong , et al., “The Life‐Course Changes in Muscle Mass Using Dual‐Energy X‐Ray Absorptiometry: The China BCL Study and the US NHANES Study,” Journal of Cachexia, Sarcopenia and Muscle (2024), Advance online publication, 10.1002/jcsm.13522. PMC11446696 38952048

[2] I. Kwiatkowska , J. Olszak , P. Formanowicz , and D. Formanowicz , “Nutritional Status and Habits Among People on Vegan, Lacto/Ovo‐Vegetarian, Pescatarian and Traditional Diets,” Nutrients 14, no. 21 (2022): 4591. 36364853 10.3390/nu14214591 PMC9657343

[3] Y. Zhang , X. Liu , Y. Ma , and X. Li , “Physical Activity, Sedentary Behavior, Fruit and Vegetable Consumption, and Sarcopenia in Older Chinese Adults: A Cross‐Sectional Study,” Nutrients 15, no. 15 (2023): 3417. 37571354 10.3390/nu15153417 PMC10420903

[4] Z. Li , X. Tong , Y. Ma , T. Bao , and J. Yue , “Prevalence of Depression in Patients With Sarcopenia and Correlation Between the Two Diseases: Systematic Review and Meta‐Analysis,” Journal of Cachexia, Sarcopenia and Muscle 13, no. 1 (2022): 128–144. 34997702 10.1002/jcsm.12908 PMC8818614

[5] H. Wan , Y. H. Hu , W. P. Li , et al., “Quality of Life, Household Income, and Dietary Habits Are Associated With the Risk of Sarcopenia Among the Chinese Elderly,” Aging Clinical and Experimental Research 36, no. 1 (2024): 29. 38334908 10.1007/s40520-023-02656-9 PMC10857955

[6] S. Roberts , P. Collins , and M. Rattray , “Identifying and Managing Malnutrition, Frailty and Sarcopenia in the Community: A Narrative Review,” Nutrients 13, no. 7 (2021): 2316. 34371823 10.3390/nu13072316 PMC8308465

